# Characterization of a Novel Aspartic Protease from *Rhizomucor miehei* Expressed in *Aspergillus niger* and Its Application in Production of ACE-Inhibitory Peptides

**DOI:** 10.3390/foods10122949

**Published:** 2021-11-30

**Authors:** Shounan Wang, Peng Zhang, Yibin Xue, Qiaojuan Yan, Xue Li, Zhengqiang Jiang

**Affiliations:** 1 Department of Nutrition and Health, College of Food Science and Nutritional Engineering, China Agricultural University, Beijing 100083, China; wshounan@cau.edu.cn (S.W.); b20193060539@cau.edu.cn (Y.X.); 2 Key Laboratory of Food Bioengineering (China National Light Industry), College of Engineering, China Agricultural University, Beijing 100083, China; zp2016@cau.edu.cn (P.Z.); lixuel@cau.edu.cn (X.L.)

**Keywords:** aspartic protease, milk-clotting, *Rhizomucor miehei*, *Aspergillus niger*, ACE-inhibitory peptides

## Abstract

*Rhizomucor miehei* is an important fungus that produces aspartic proteases suitable for cheese processing. In this study, a novel aspartic protease gene (*RmproB*) was cloned from *R. miehei* CAU432 and expressed in *Aspergillus niger*. The amino acid sequence of RmproB shared the highest identity of 58.2% with the saccharopepsin PEP4 from *Saccharomyces cerevisiae*. High protease activity of 1242.2 U/mL was obtained through high density fermentation in 5 L fermentor. RmproB showed the optimal activity at pH 2.5 and 40 °C, respectively. It was stable within pH 1.5–6.5 and up to 45 °C. RmproB exhibited broad substrate specificity and had *K*_m_ values of 3.16, 5.88, 5.43, and 1.56 mg/mL for casein, hemoglobin, myoglobin, and bovine serum albumin, respectively. RmproB also showed remarkable milk-clotting activity of 3894.1 SU/mg and identified the cleavage of Lys21-Ile22, Leu32-Ser33, Lys63-Pro64, Leu79-Ser80, Phe105-Met106, and Asp148-Ser149 bonds in κ-casein. Moreover, duck hemoglobin was hydrolyzed by RmproB to prepare angiotensin-I-converting enzyme (ACE) inhibitory peptides with high ACE-inhibitory activity (IC_50_ of 0.195 mg/mL). The duck hemoglobin peptides were further produced at kilo-scale with a yield of 62.5%. High-level expression and favorable biochemical characterization of RmproB make it a promising candidate for cheese processing and production of ACE-inhibitory peptides.

## 1. Introduction

Proteases can hydrolyze proteins through the cleavage of peptide bonds, thus having an important position in industrial enzymes [1]. Aspartic proteases (EC 3.4.23.X) are a class of proteases with an optimal activity in acidic environments and have wide industrial applications, particularly for food and feed [2,3]. Chymosin is a type of neonatal gastric aspartic proteases. The chymosin from calf stomach has been used to produce various cheeses [4]. It is the best milk coagulant owing to the high hydrolysis specificity against κ-casein at the position of Phe105-Met106 [5]. With the increase of cheese consumption, the demand for chymosin is rapidly rising. Many studies have focused on novel proteases as substitutes for bovine chymosin [1]. Microbial proteases are devoted as one of the promising substitutes for the bovine chymosin [6]. Especially the milk-clotting proteases from *Rhizomucor miehei*, *R. pusillus* and *Mucor mucedo* have widely been applied in the industries [7,8,9]. Meanwhile, novel proteases from these fungi and efficient expression have potential application for cheese production.

Genetic engineering technology is a promising approach to discover and produce novel proteases. *Aspergillus niger* has been demonstrated as an important host that produces various proteins based on its high protein secretion efficiency, the ability of complex post-translational modifications, and excellent safety [10,11]. As a non-pathogenic microorganism, most *A. niger* strains’ cultures do not contain mycotoxin and are classified into GRAS (Generally Recognized As Safe) by FDA [12]. Advances in eukaryotic genetic technologies have promoted to express various proteins in *A. niger*. Many attempts have been contributed to hyperexpress bovine chymosin in *A. niger* through multiple strategies [13,14]. The secretion of bovine chymosin in *A. niger* was improved by fusion expression with glucoamylase gene (*glaA*) [15]. Nitrosoguanidine (NTG) mutagenesis and robotic screening program were performed to increase the production of recombinant bovine chymosin [16]. Another approach was to improve the N-glycosylation site of the prochymosin. Compared with the native chymosin, the yield of highly glycosylated chymosin was increased by more than 100% [17]. To date, the recombinant bovine chymosin produced by *A. niger* var. *awamori* has been commercially used in cheese industry [4]. However, the expression efficiency of heterologous proteins is still restricted by the protein degradation, inefficient transcription, incorrect protein folding, and the difficulty in controlling of mycelial morphology during fermentation, thus hampering the efficient utilization of *A. niger* [18,19]. So far, no reports have been studied on the expression of *Rhizomucor* spp. proteases in *A. niger*.

Bioactive peptides are short amino-acid sequences with different biological properties, which have a positive effect on human health. Peptides derived from food proteins with angiotensin-I-converting enzyme (ACE) inhibitory activity are considered as natural supplements to regulate blood pressure without side effects [20]. Protease hydrolysis is commonly used method to prepare ACE-inhibitory peptides from food proteins. At present, the production of antihypertensive hydrolysates using commercial proteases has been abundantly investigated [21]. The application of novel proteases to prepare bioactive peptides will attract more attention.

*R. miehei* is an important fungus that has produced multiple proteases and lipases [22,23]. In the previous study, we have heterologously expressed an aspartic protease (RmproA) from *R. miehei* in *P. pastoris* [24]. RmproA showed potential application for preparation of turtle peptides and meat tenderization. In this study, to explore more proteases suitable for the food industry, a novel aspartic protease (RmproB) from *R.miehei* was heterologously expressed in *A. niger*. Moreover, RmproB was purified and its biochemical properties were characterized, as well as the potential applications for milk-clotting and production of ACE-inhibitory peptides.

## 2. Materials and Methods

### 2.1. Strains, Culture Media, and Reagents

*R. miehei* CAU432 has been deposited in the China General Microbiological Culture Collection Center (CGMCC No. 4967) with the whole genome sequenced [24]. *Escherichia coli* Trans5α (Transgene, Beijing, China) was used for molecular cloning. *A. niger* strain FBL-A (*ΔglaA*) was used for expression of the protease. It was cultured in Czapek-Dox medium. If necessary, 200 μg/mL hygromycin was added for the hygB selection marker. Casein, hemoglobin, gelatin, whey protein, lactoglobulin, bovine serum albumin, myoglobin, egg albumin, protamine sulfate, collagen, phenylmethylsulfonyl fluoride (PMSF), and pepstatin A were obtained from Sigma-Aldrich Corporation (Darmstadt, Germany). Other chemicals used in this study were provided by Biodee Corporation (Beijing, China).

### 2.2. Bioinformatics Analysis and Construction of the Recombinant Plasmid

Multiple amino acid sequence alignment of RmproB was used by CLUSTALW (https://www.genome.jp/tools-bin/clustalw, accessed on 20 April 2021). The gene of *RmproB* (GenBank: MZ547666) was PCR amplified from cDNA of *R. miehei* CAU432. To increase the secretion capacity of RmproB, its signal peptide was replaced by the signal peptide of glucoamylase (*glaA*) from *A. niger*. The putative signal peptide sequence of RmproB was predicted by the SignalP 5.0 (http://www.cbs.dtu.dk/services/SignalP/, accessed on 10 May 2021). The *A. niger* glucoamylase promoter P*_glaA_* and terminator T*_glaA_* gene were PCR amplified with genomic DNA of *A. niger* FBL-A. The protease expression cassette P*_glaA_*-*RmproB*-T*_trpC_* and hygromycin B resistance gene cassette P*_gpda_*-*hygB*-T*_glaA_* were assembled using the ClonExpress MultiS One Step Cloning Kit (Vazyme, Nanjing, China) and cloned into pEASY-Blunt cloning vector (TransGen, Beijing, China) to construct the expression plasmid pRmproB. All primers were listed in Supplementary Table S1.

### 2.3. Transformation and Screening of the Recombinant A. niger

The *A. niger* strain and linearized plasmid pRmproB were transformed using the protoplast transformation method with 200 μg/mL hygromycin for selection [25]. The transformants were subcultivated on CD (Czapek-Dox medium) agar plates and confirmed by PCR analysis and sequencing. Positive transformants with correct gene insertion were subjected to shake flask fermentation.

### 2.4. Expression and Production of RmproB

The medium and fermentation conditions used for recombinant protein expression were described by Cai et al. [26]. The transformants were inoculated in 50 mL fermentation medium under shaking at 250 rpm for 5 days. The culture medium was centrifuged at 10,000 *g* (4 °C and 15 min) to collect crude enzyme. The transformant with the highest protease activity was used to high density fermentation.

High density fermentation was performed in BIOTECH-5BG fermentor (Bxbio, China) which contained 3 L fermentation medium comprising corn starch hydrolysate (4%, *w/v*), peptone (2%, *w/v*), yeast extract (1%, *w/v*), and KH_2_PO_4_ (0.5%, *w/v*). The pH was adjusted to pH 6.0 with 28.0% aqueous ammonia and 10% phosphoric acid. The fermentation of recombinant *A. niger* consisted of two stages: The first stage was the hyphal growth phase with the agitation rate and temperature kept at 300 rpm and 30 °C, respectively. With the growth of mycelium, the dissolved oxygen (DO) decreased continuously. When the starch hydrolysate in the fermentation medium was completely consumed (about 60−72 h), the first stage was ended. For the second stage of enzyme production phase, corn starch hydrolysate (50%, *w/v*) was fed into initial fermentation volume at 5 mL/h/L. The DO was kept above 5% by controlling the agitation rate and air flow until the end of fermentation. During the fermentation process, the wet cell weight, protein concentration, and protease activity were monitored.

### 2.5. Measurement of Protease Activity and Protein Content

The Folin-phenol method previously described by Anson was applied for protease activity measurement [27]. Casein was dissolved in lactate buffer (50 mM and pH 2.5) at 1.0% (*w/v*) and used as substrate. One unit (U) of protease activity was defined as the amount of protease that is needed for substrate hydrolysis and releasing of 1 μg tyrosine per minute. The standard curve of tyrosine was exhibited in Supplementary Figure S1. The Lowry method was used to determine the protein concentration with bovine serum albumin (Sigma, Darmstadt, Germany) as the standard [28].

### 2.6. Purification of RmproB

The crude enzyme was dialyzed overnight in 20 mM sodium phosphate buffer (PBS, pH 6.0). The dialysate was collected and purified using Q-Sepharose Fast Flow column that pre-equilibrated with PBS. PBS containing NaCl concentration of 0–500 mM was used as the eluent and the enzyme was eluted at 1 mL/min. The fraction with the protease activity was concentrated and further separated through gel-filtration using Sephacryl-100 column (1.0 ×100 cm). PBS was used to elute the protease at 0.3 mL/min. The purity of protease was analyzed by SDS-PAGE.

### 2.7. Biochemical Properties of RmproB

The optimal pH of RmproB for activity was investigated at 40 °C using 1.0% (*w*/*v*) casein as the substrate in 50 mM different buffers. The buffers used were KCl-HCl (pH 1.0–2.0), lactate (pH 2.0–4.0), citrate (pH 3.5–6.5), phosphate (pH 6.5–8.0), and Tris-HCl (pH 8.0–9.0). To determine the pH stability, the enzyme was pre-incubated in various pH buffers at 40 °C for 30 min. The residual protease activity was measured by the standard assay using 1% casein as a substrate. The optimal temperature of RmproB was determined at temperatures ranging from 20 °C to 60 °C in 50 mM lactate buffer (pH 2.5). Thermostability was investigated by assessing the residual protease activity after pre-incubation at 25–60 °C for 30 min.

Effects of metal ions and chemical reagents on the protease activity of RmproB were tested. After incubation with 50 mM lactate buffer (pH 2.5) containing 1 mM K^+^, Na^+^, Ag^+^, Zn^2+^, Mg^2+^, Fe^2+^, Ca^2+^, Ni^2+^, Cu^2+^, Co^2+^, Cr^2+^, Mn^2+^, Ba^2+^, Fe^3+^, SDS, or β-mercaptoethanol at 40 °C for 30 min, the residual protease activity of RmproB was measured with casein (1%, *w*/*v*) as the substrate. Protease inhibitors of pepstatin A (aspartic protease inhibitor, 0.01 mM and 0.05 mM), iodoacetamide (cysteine protease inhibitor, 1.0 mM and 5.0 mM), PMSF (serine protease inhibitor, 1.0 mM and 5.0 mM), and EDTA (metalloprotease inhibitor, 1.0 mM and 5.0 mM) were selected and their effects on protease activity of RmproB were determined.

### 2.8. Substrate Specificity and Kinetic Parameters of RmproB

The substrate specificity of RmproB was determined using casein, hemoglobin, collagen, skimmed milk, whey protein, lactoglobulin, bovine serum albumin, myoglobin, egg albumin, protamine sulfate, soy protein isolate, or gelatin as the substrate. The protease activity was assayed in 50 mM lactate buffer (pH 2.5) containing 10 mg/mL of each substrate and kept at 40 °C for 10 min. The specific activity was measured by the protease activity per mg protein.

The kinetic parameters of RmproB for casein, hemoglobin, myoglobin, and bovine serum albumin were measured in 50 mM lactate buffer (pH 2.5) at 40 °C for 5 min. *K*_m_ and *V*_max_ values were calculated using the software GraFit.

### 2.9. Analysis of Milk-Clotting Activity and Casein Hydrolysis of RmproB

Milk-clotting activity of RmproB was measured according to the previously reported method with minor modifications [29]. Skim milk powder (10.0%, *w*/*v*) was dissolved in PBS (50 Mm, pH 4.0) containing 5 mM CaCl_2_ and used as the substrate. Then, aliquots of the substrate (5 mL) were mixed with the RmproB solution (0.5 mL) and kept at 37 °C under manual rotation. The clotting time was recorded after coagulation was observed. The milk-clotting activity (Soxhlet units, SU) was defined as the amount of enzyme needed to clot 1 mL substrate in 40 min at 37 °C.

The effects of pH and CaCl_2_ on the milk-clotting activity of RmproB were investigated. RmproB was added to the 10% skim milk powder in citrate buffer within pH 3.5–6.5. The milk-clotting activity of RmproB at pH 4.0 was taken as 100%. The milk-clotting index viz. the ratio of milk-clotting activity to protease activity (CA/PA) was calculated. The effects of CaCl_2_ on milk-clotting activity of RmproB were evaluated using 10% skim milk powder in citrate buffer (pH 4.0) containing different CaCl_2_ concentrations. The milk-clotting activity at 5 mM CaCl_2_ was taken as 100%.

To analyze the casein hydrolysis by RmproB, commercial bovine caseins of α_s_-, β-, and κ-casein (1.0%, *w*/*v*) were dissolved in 50 mM citrate buffer (pH 4.0) and mixed with RmproB (10 SU/mL). The reaction was kept at 40 °C and samples were taken at time intervals of 0, 5, 10, and 30 min. After enzyme deactivation through boiling for 5 min, hydrolysates of casein were analyzed by SDS-PAGE. The cleavage sites of RmproB in κ-casein were analyzed by MALDI-TOF MS (matrix-assisted laser desorption ionization-time of flight mass spectrometry). Peptide sequence retrieval of κ-casein hydrolysates was conducted using MASCOT (http://www.matrixscience.com, accessed on 20 June 2021) as previously described [30].

### 2.10. Preparation of ACE-Inhibitory Peptides by RmproB

The duck hemoglobin (10%, *w*/*v*) was suspended in 50 mM lactate buffer (pH 2.5). RmproB (50 U/mL) was added and hydrolyzed at 40 °C for 0, 2, 4, 6, and 8 h. Then, the suspension was boiled for 5 min to deactivate the enzyme. After centrifugation at 10,000× *g* for 10 min, the supernatants were analyzed for the molecular weight distribution and the ACE-inhibitory activity.

Molecular weight distributions of the duck hemoglobin and hydrolysates were analyzed by HPLC (high-performance liquid chromatography) system using the method described by Zhang et al. [31]. In short, the mobile phase was deionized water, acetonitrile, and trichloroacetic acid with a ratio of 55:45:0.1. The suspension of 5 mg/mL duck hemoglobin or the lyophilized hydrolysates was filtered and injected into an Agilent 1260 HPLC system with TSKgel-G2000SWXL column (7.8 × 300 mm). The flow rate was 0.5 mL/min, and the absorbance was set on 214 nm. Nine standard proteins (glycine-tyrosine, valine-tyrosine-valine, cytochrome C, apomyoglobin, ribonuclease A, holo-transferrin, angiotensin II, leucine encephalin, and methionine enkephalin) were used to plot the calibration curves of molecular weight. The chromatogram was divided into four areas with different molecular weight (<1 kDa, 1–2 kDa, 2–5 kDa, and >5 kDa). The molecular weight distribution of the peptide fractions was determined as the percentage of the total area.

The ACE-inhibitory activity of duck hemoglobin peptides with different hydrolysis times was using the previously reported method [32]. The ACE-inhibitory activity (%) was calculated based on the following equation:

(1) $$\mathrm{ACE}-\mathrm{inhibitory}\;\mathrm{activity}\;(\%)=\frac{\mathrm{Absorbance}\;\mathrm{of}\;\mathrm{control}\;-\;\mathrm{Absorbance}\;\mathrm{of}\;\mathrm{sample}}{\mathrm{Absorbance}\;\mathrm{of}\;\mathrm{control}\;-\;\mathrm{Absorbance}\;\mathrm{of}\;\mathrm{blank}}\times \;100\%$$

The duck hemoglobin peptides were further produced at the kilo-scale in an agitated reactor (YHL-20L, Yuhua, Zhucheng, China). The duck hemoglobin (10%, *w*/*v*) suspended with 10 L pure water was added in the reactor. The pH was adjusted to pH 2.5 with HCl. RmproB (50 U/mL) was added and the hydrolysis process was kept at 40 °C with a stirring rate of 200 rpm. After enzymatic hydrolysis for 8 h, the hydrolysate was conducted using a plate filter (WBG-1, Hundong, Guangzhou, China) with a 0.45 μm filter cloth (300 mm) for the solid–liquid separation. The liquid fraction was concentrated by vacuum rotary evaporation (R-1050, Ensheng, Shanghai, China) and then spray-dried by spray dryer (PGL-B, Changzhoujiafa, Jiangsu, China).

## 3. Results

### 3.1. Gene Cloning and Sequence Analysis of RmproB

An aspartic protease gene (*RmproB*) from *R. miehei* was cloned. The ORF (open reading frame) of the gene (*RmproB*) was 1779 bp containing five introns (228 bp, 69 bp, 60 bp, 56 bp, and 46 bp) and encoded a protein of 439 amino acids with a predicted signal peptide of 19 amino acids. The gene sequence was submitted into the GenBank database under the accession number MZ547666.

**Figure 1:**
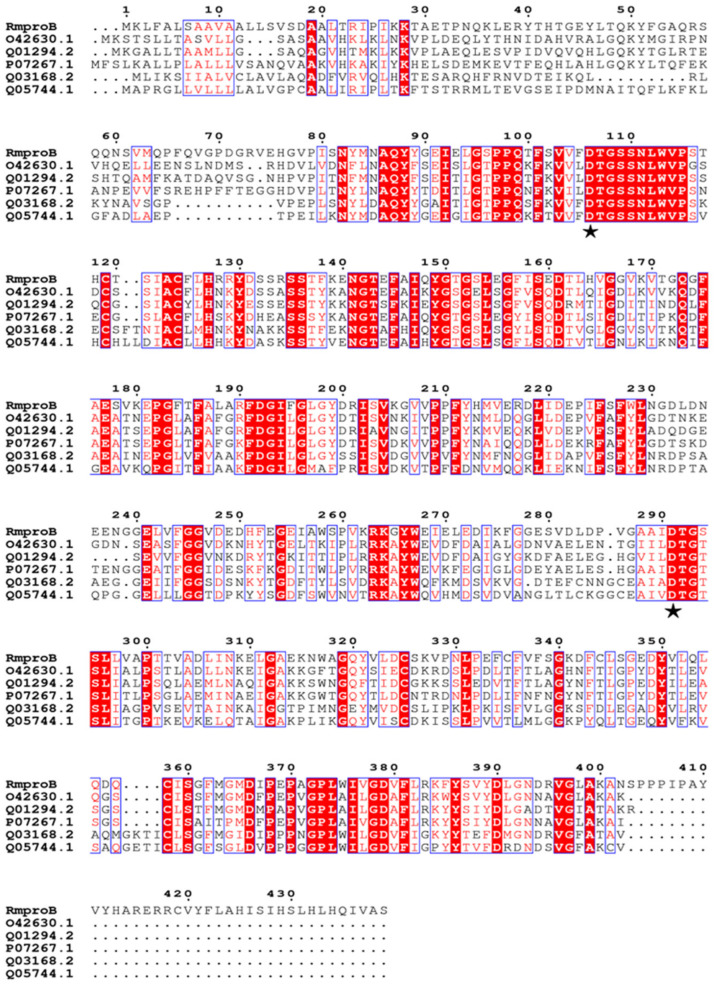
Multiple amino acid sequence alignment of RmproB with other A1 family aspartic proteases. RmproB was the aspartic protease from *R. miehei* in this study. The sequences, O42630.1 (*Aspergillus fumigatus*), Q01294.2 (*Neurospora crassa*), P07267.1 (*Saccharomyces cerevisiae*), Q03168.2 (*Aedes aegypti*), and Q05744.1 (*Gallus gallus*) were downloaded from the NCBI protein database. Identical residues are shaded in red, and conserved residues are shown in blue boxes. The conversed catalytic residues are marked with asterisks.

According to the multiple amino acid sequence alignments (Figure 1), RmproB showed the highest amino acid sequence identity of 58.2% with the saccharopepsin PEP4 from *Saccharomyces cerevisiae* (P07267.1) [33], followed by the aspartic proteinase PEP2 from *A. fumigatus* (50.1%, O42630.1) [34], and the vacuolar protease A from *Neurospora crassa* (47.9%, Q01294.2) [35]. Especially, it shared very low amino acid sequence identity of 18.9% with RmproA from *R. miehei* (ATY35192.1) [24]. Asp106 and Asp291 in RmproB were the putative conversed catalytic residues.

### 3.2. Expression and Purification of RmproB

**Figure 2:**
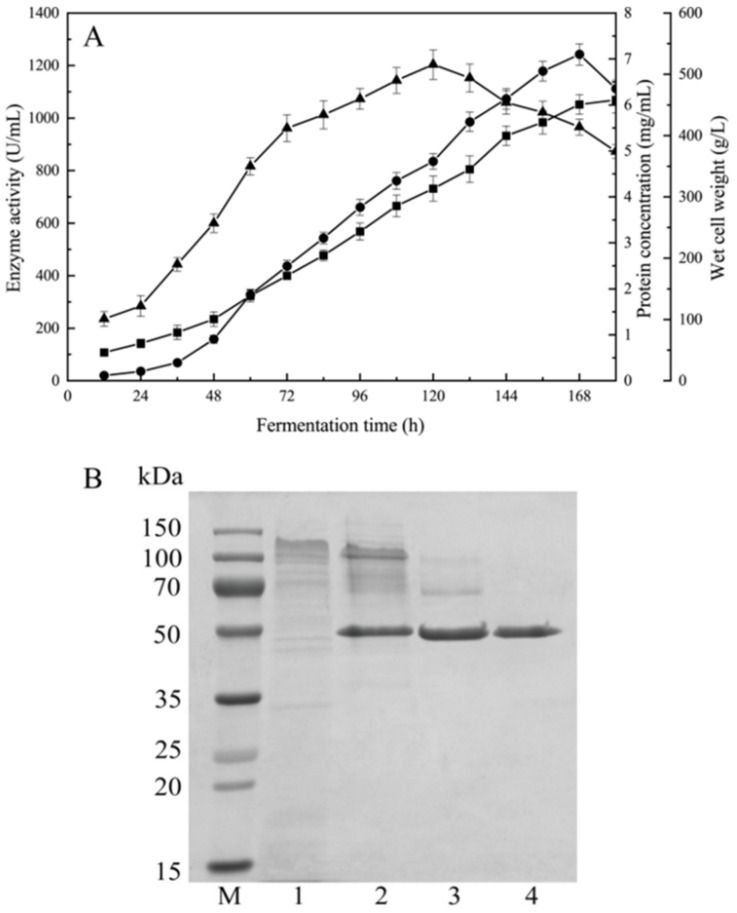
Time-course of RmproB produced by *A. niger* in 5 L fermentor (**A**) and SDS-PAGE analysis of the purified RmproB (**B**). Symbols are enzyme activity (●), protein concentration (■), and wet cell weight (▲) of high-density fermentation (**A**). Lane M, protein molecular weight marker; lane 1, *A. niger* FBL-A for control; lane 2, the crude supernatant; lane 3, RmproB after QSFF; lane 4, RmproB after S-100 (**B**).

RmproB was expressed under the control of promoter P*_glaA_* and secreted into the medium owing to the glucoamylase signal peptide. The protease was successfully expressed as the main extracellular protein in the fermentation broth. The highest protease activity of 1242.2 U/mL with protein concentration of 6.1 mg/mL was produced after incubation for 168 h in 5 L fermenter (Figure 2A).

After Q-Sepharose chromatography and Sephacryl-100 gel filtration chromatography, RmproB was purified 3.9 folds to apparent homogeneity with a recovery yield of 18.8%. The purified RmproB exhibited specific activity of 3176.1 U/mg (Supplementary Table S2). The molecular weight of purified RmproB was estimated to be 49.4 kDa with a single band in SDS-PAGE (Figure 2B).

### 3.3. Biochemical Characterization of RmproB

**Figure 3:**
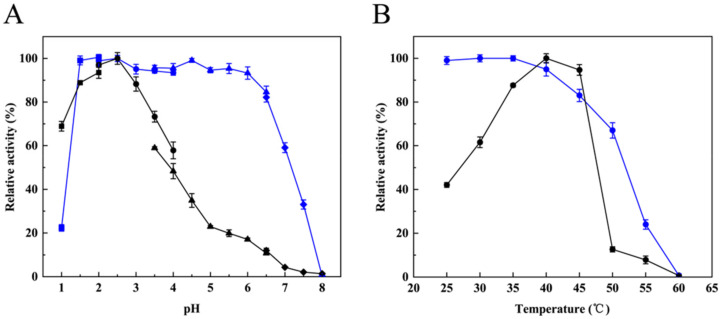
Optimal pH (**A**, black polyline) and pH stability (**A**, blue polyline) of RmproB, optimal temperature (**B**, black polyline) and thermostability (**B**, blue polyline) of RmproB. The optimal pH of RmproB was determined in 50 mM various buffers at 40 °C. The buffers used are KCl-HCl (■, pH 1.0–2.0); lactate (●, pH 2.0–4.0); citrate (▲, pH 3.5–6.5); phosphate (◆, pH 6.5–8.0). The pH stability was assessed by measuring the residual protease activity after the enzyme was pre-incubated at 40 °C for 30 min in buffers mentioned above. The optimal temperature was tested in 50 mM lactate buffer (pH 2.5) at different temperatures (20–60 °C). The thermostability was evaluated by investigating the residual protease activity after pre-incubation in 50 mM lactate buffer (pH 2.5) at 25–60 °C for 30 min.

The optimal pH of RmproB was pH 2.5 in lactate buffer (Figure 3A). The protease exhibited high stability within an acid pH range of 1.5–6.5 (activity above 80%), and 99.1% residual activity was retained after incubation at pH 1.5 for 30 min (Figure 3A). RmproB showed optimal activity at 40 °C and was stable up to 45 °C for 30 min, maintaining around 83% residual activity (Figure 3B).

The protease activity of RmproB was activated by Mn^2+^ (120.5%) and strongly inhibited by SDS (4.4%). The other chemicals exhibited no or slight effects on the protease activity of RmproB. The enzyme activity of RmproB was completely inhibited by 0.05 mM pepstatin A, suggesting that RmproB is an aspartic protease (Supplementary Table S3). Protease inhibitors of PMSF, EDTA, and iodoacetamide had no significant effects on the protease activity of RmproB, indicating that the serine residue, metal ions, or -SH group had no effect on the protease activity of RmproB.

The purified RmproB showed broad substrate specificity towards the tested substrates (Table 1). RmproB exhibited the highest protease activity towards casein (100.0%), followed by hemoglobin (94.8%), myoglobin (80.4%), bovine serum albumin (73.8%), and skimmed milk (60.4%). However, it did not exhibit any detectable activity towards soy protein isolate, gelatin, protamine sulfate, and collagen.

The kinetic parameters of RmproB for casein, hemoglobin, myoglobin, and bovine serum albumin were determined. The *K*_m_ and *V*_max_ values of RmproB were 3.16 mg/mL and 3583 μmol/min/mg for casein, 5.88 mg/mL and 4572 μmol/min/mg for hemoglobin, 5.43 mg/mL and 3623 μmol/min/mg for myoglobin, and 1.56 mg/mL and 1732 μmol/min/mg for bovine serum albumin.

### 3.4. Milk-Clotting Activity and Casein Hydrolysis

The milk-clotting activity of crude enzyme was measured to be 1285.7 SU/mL. After purification, RmproB exhibited high milk-clotting activity of 3894.1 SU/mg. It showed the optimal milk-clotting activity at pH 4.0 and 5 mM CaCl_2_ (Supplementary Figure S2). Under the optimal conditions, RmproB exhibited the milk-clotting index (CA/PA) of 6.1.

**Figure 4:**
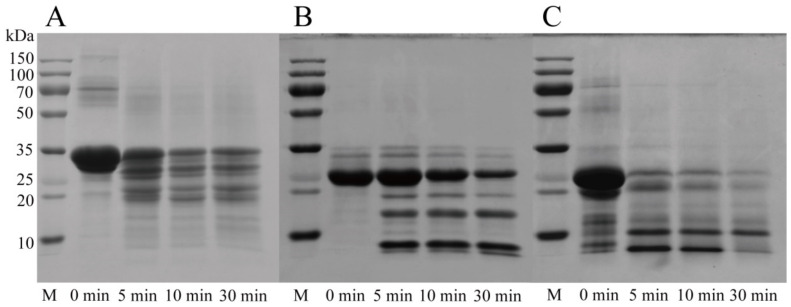
SDS-PAGE analysis of α_s_-casein (**A**), β-casein (**B**), and κ-casein (**C**) hydrolyzed by RmproB. Lane M, protein molecular weight marker; other lanes, three types of caseins hydrolyzed by RmproB (10 SU/mL) in 50 mM pH 4.0 citrate buffer for 0, 5, 10, and 30 min.

Multiple caseins were hydrolyzed by RmproB. The protease exhibited various hydrolysis patterns towards three different components of casein. SDS-PAGE analysis revealed that RmproB showed higher hydrolysis ability against κ-casein than those of α_s_-casein and β-casein (Figure 4). After incubation for 5 min, κ-casein was hydrolyzed into two major peptide fragments with molecular weight of 8 kDa and 10 kDa. In contrast, α_s_-casein was randomly and partially hydrolyzed into small fragments with molecular weights of 18–30 kDa. β-Casein was only degraded into two major peptide fragments with molecular weight of 8 kDa and 15 kDa. Based on the peptides from κ-casein hydrolyzed by RmproB, Lys21-Ile22, Leu32-Ser33, Lys63-Pro64, Leu79-Ser80, Phe105-Met106, and Asp148-Ser149 bonds in κ-casein were identified as the primary cleavage sites (Table 2).

### 3.5. Preparation of ACE-Inhibitory Peptides by RmproB

**Figure 5:**
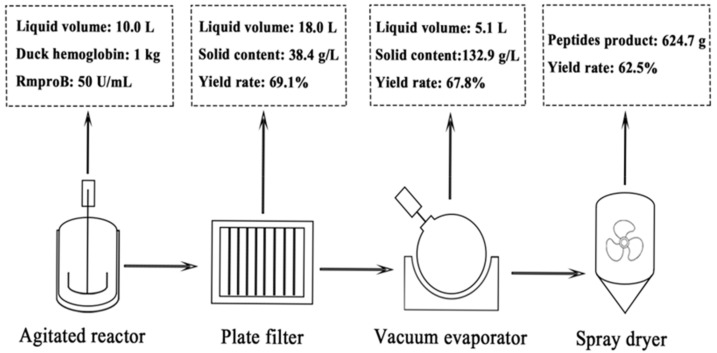
Flow diagram of production of duck hemoglobin peptides at kilo-scale. The solid content was defined as the ratio of the weight of the lyophilized solid to the total solution volume. The yield was defined as the percentage of the weight of the obtained duck hemoglobin peptides to the initial substrate.

The potential of RmproB was evaluated to prepare ACE-inhibitory peptides from duck hemoglobin. The molecular weight distribution and ACE-inhibitory activity of the hydrolysates at different hydrolysis intervals were shown in Supplementary Figure S3 and Table 3. With the increase of hydrolysis time, duck hemoglobin was hydrolyzed into smaller peptides (<1 kDa) from the intact molecule (>5 kDa). Small peptides (<1 kDa) were generated at a large amount of 88.5%. After 8 h hydrolysis, duck hemoglobin peptides showed high ACE-inhibitory activity of 90.7% at concentration of 0.5 mg/mL. The IC_50_ value of duck hemoglobin peptides for ACE-inhibitory activity was 0.195 mg/mL. The production of duck hemoglobin peptides was enlarged to kilo-scale, as shown in the flow diagram (Figure 5). After the four-step process, 624.7 g peptides powder was obtained with a product yield of 62.5%.

## 4. Discussion

Aspartic proteases have a wide range of applications in various food fields, including cheese, bakery, and wine [1]. *R. miehei* has been reported to produce multiple proteases and lipases for food application, such as cheese processing, meat tenderization, and lipid synthesis [22,23]. Much work has been done to study the wild type aspartic proteases from *R. miehei* to improve the volatile composition and sensory properties of cheese [23,36]. In this study, a novel aspartic protease (RmproB) from *R. miehei* CAU432 was extracellularly expressed in *A. niger*. RmproB exhibited the highest sequence similarity of 58.2% with the saccharopepsin PEP4 from *S. cerevisiae* (Figure 1). Combined with the inhibition by pepstatin A, RmproB should be a new A1 family aspartic protease [33].

Usually, proteins expressed in *A. niger* are mainly achieved through using organism-specific, endogenous gene expression elements (endogenous transcription factors, signal peptides, promoters, and terminators) [11]. The wild type glucoamylase and amylase secreted by *A. niger* have strong promoters P*_glaA_* and P*_amyA_*, which are often used to regulate the expression of heterologous proteins [19]. RmproB was expressed under the regulation of the promoter P*_glaA_* and the glucoamylase signal peptide. The highest protease activity of RmproB (1242.2 U/mL) was produced in 5 L fermentor (Figure 2A), which is much higher than those microbial aspartic proteases from *Trichoderma harzianum* (321.8 U/mL) [37] and *Penicillium* sp. (89.3 U/mL) [38] expressed in *P. pastoris* but lower than the acid protease from *A. kawachii* expressed in *A. niger* (5543 U/mL) [39] and RmproA from *R. miehei* expressed in *P. pastoris* (3480.4 U/mL) [24].

The molecular weight of aspartic proteases commonly ranges from 35 to 50 kDa [3]. RmproB had a molecular weight of 49.4 kDa (Figure 2B), which is similar to that of RmproA from *R. miehei* CAU432 (50.6 kDa) [24] and higher than the wild type aspartic proteases from *R. miehei* isolated from decaying wood (37 kDa) [40]. The purified RmproB showed specific activity of 3176.1 U/mg (Supplementary Table S2), which is much higher than those of the aspartic proteases from *Talaromyces leycettanus* (1795.4 U/mg) [41] and RmproA (773.3 U/mg) [24]. The optimal pH of RmproB is pH 2.5 (Figure 3A), which is the same as the recombinant aspartic protease from *T. harzianum* (pH 2.5) [37] but lower than the wild type aspartic protease from *A. niger* (pH 3.5) [42], the milk-clotting protease from *R. miehei* NRRL 3500 (pH 4.1) [43], and the RmproA from *R. miehei* CAU432 (pH 5.5) [24]. Furthermore, RmproB exhibited the optimal temperature at 40 °C (Figure 3B), which is higher than the milk-clotting protease from *R. miehei* NRRL 3500 (35 °C) [43] and lower than the wild type aspartic peptidase from *R. miehei* (55 °C) [40] and the RmproA from *R. miehei* CAU432 (55 °C) [24]. RmproB lost its activity after incubation at 60 °C for 30 min (Figure 3B), indicating the ability for thermal inactivation of RmproB to prevent excessive hydrolysis of substrates. Extensive or unspecific hydrolysis towards caseins may lead to undesirable characteristics of cheese [5]. Thermal inactivation can prevent residual protease during manufacturing and avoid undesirable texture and flavor in the cheese [44]. RmproB exhibited broad substrate specificity towards casein, hemoglobin, myoglobin, and bovine serum albumin, with *K*_m_ values of 3.16, 5.88, 5.43, and 1.56 mg/mL, respectively. The *K*_m_ values of RmproB for different substrates are lower than that of MpAPr1 from the yeast *Metschnikowia pulcherrima* (5.9 mg/mL towards casein) [45], aspergillopepsin A-like from *A. niger* (6.3 mg/mL towards hemoglobin) [42], and the aspartic protease from *A. oryzae* MTCC 5341 (8 mg/mL towards hemoglobin) [46].

RmproB showed high milk-clotting activity of 3894.1 SU/mg, which is similar to the chymosin from *Rhizopus microsporus* var. *rhizopodiformis* (3905 SU/mg) [47] and higher than those from *A. oryzae* MTCC 5341 (3500 SU/mg) [48] and *Termitomyces clypeatus* MTCC 5091 (150.9 SU/mg) [44]. High milk-clotting activity and low protease activity are ideal properties of proteases being suitable for cheese production [6]. RmproB showed the optimal coagulation activity at pH 4.0 with a high milk-clotting index (CA/PA) of 6.1 (Supplementary Figure S2A). However, the milk-clotting protease from *R. miehei* NRRL 3500 showed optimal pH at 5.6 with CA/PA of 2.3 [43]. For commercial bovine chymosin, high concentration of CaCl_2_ causes an increase in both milk-clotting activity and the total protease activity [4]. RmproB showed the optimal milk-clotting activity at 5 mM CaCl_2_ concentration (Supplementary Figure S2B), which avoids the growing risk of protease activity due to the addition of calcium ions. The hydrolysis capacity at a specific position in the κ-casein is a crucial factor for the assessment of coagulation efficiency. The bovine chymosin specifically cleaves κ-casein at Phe105-Met106 residues and releases the C-terminal region of κ-casein (caseinomacropeptide), causing a decrease in the colloidal stability of micelles and resulting in milk curdling [6]. RmproB showed a significant degradation of κ-casein but a relatively weak degradation of α_s_-casein and β-casein (Figure 4). Similar properties of milk-clotting proteases have been demonstrated from *Bacillus amyloliquefaciens* GSBa-1 [49] and *T. clypeatus* MTCC 5091 [44]. SDS-PAGE exhibited that κ-casein was hydrolyzed by RmproB, yielding two major peptide fragments with molecular weights of 8 kDa and 10 kDa (Figure 4). The peptide bonds of Lys21-Ile22, Leu32-Ser33, Lys63-Pro64, Leu79-Ser80, Phe105-Met106, and Asp148-Ser149 in κ-casein were the primary hydrolysis sites for RmproB (Table 2). The mechanism of milk coagulation for RmproB is similar to the bovine chymosin. The cleavage of Phe105-Met106 bond may be the initial and critical step during coagulation. The other cleavage sites can be attributable to the secondary proteolysis of peptides [44].

Duck meat is consumed in large quantities all over the world, especially in Asia. In 2019, the number of commercial ducks sold was up to 4.3 billion in China [50]. Duck blood is one of the main by-products during the slaughter process. As an abundant source of protein, duck blood is considered a non-allergenic protein compared with soy and dairy proteins [51]. Recently, studies have discovered antioxidant peptides from duck blood [51,52]. Food-derived ACE-inhibitory peptides are promising components for the prevention and treatment of hypertension [53]. Turtle meat was hydrolyzed by RmproA to prepare ACE-inhibitory peptides with ACE-inhibitory activity of 88% at concentration of 1 mg/mL [24]. In this study, duck hemoglobin hydrolyzed by RmproB showed high ACE-inhibitory activity of 90.7% at concentration of 0.5 mg/mL (Table 3). The IC_50_ value of duck hemoglobin peptides for ACE-inhibitory activity was 0.195 mg/mL. Pepsin is the most commonly used commercial aspartic protease in the preparation of bioactive peptides. The globin derived from porcine hemoglobin was digested by pepsin to prepare ACE-inhibitory peptides with IC_50_ of 1.19 mg/mL [54]. The camel whey hydrolyzed by pepsin displayed ACE-inhibitory activity as IC_50_ of 0.197 mg/mL [55]. The pistachio hydrolysates by pepsin and trypsin showed ACE-inhibitory activity as IC_50_ of 0.87 mg/mL [56]. In kilo-scale production, the yield of duck hemoglobin peptides was up to 62.5% (Figure 5). The results verified the feasibility of RmproB used for the efficient production of ACE-inhibitory peptides from duck hemoglobin.

## 5. Conclusions

A novel A1 family aspartic protease (RmproB) from *R. miehei* was expressed in *A. niger*. High protease activity was produced through high density fermentation. RmproB showed remarkable milk-clotting activity with a high CA/PA ratio. Moreover, RmproB was used to produce bioactive peptides from duck hemoglobin. The hydrolysates show excellent ACE-inhibitory activity and satisfactory yield. The ability of milk-clotting and bioactive peptides preparation make RmproB a promising candidate for food processing. Future studies will be devoted to applying RmproB to cheese production and investigating the bioactive components of duck hemoglobin peptides.

## Figures and Tables

**Table 1:** Substrate Specific Activities of RmproB ^1^.

Substrate	Specific Activity (U/mg) ^2^	Relative Activity (%)
Casein	3176.1 ± 67.1	100
Hemoglobin	3011.1 ± 78.2	94.8 ± 2.5
Myoglobin	2552.3 ± 68.8	80.4 ± 2.2
Bovine serum albumin	2344.3 ± 79.7	73.8 ± 2.5
Skimmed milk	1917.1 ± 84.7	60.4 ± 2.7
Egg albumin	1393.5 ± 17.0	43.9 ± 0.5
Whey protein	631.3 ± 81.0	19.9 ± 2.5
Lactoglobulin	161.9 ± 148.2	5.1 ± 4.7

^1^ Specific activities are shown as mean ± SD (n = 3). The enzyme activity towards casein is defined as 100%. ^2^ Specific activities of RmproB towards the substrates were tested by the protease activity per mg protein. One unit (U) of hydrolytic activity was defined by the amount of protease that hydrolyzed substrate to release 1 μg tyrosine per minute.

**Table 2:** Identity of Peptides Derived from K-Casein Hydrolyzed by RmproB.

Start-End	Mr (expt) ^1^	Mr (calc) ^2^	Peptide Sequences ^3^
22–32	1319.7829	1319.7853	K.IAKYIPIQYVL.S
33–63	3757.8904	3757.8932	L.SRYPSYGLNYYQQKPVALINNQFLPYPYYAK.P
64–79	1776.0013	1776.0046	K.PAAVRSPAQILQWQVL.S
80–105	3012.4690	3012.4811	L.SNTVPAKSCQAQPTTMARHPHPHLSF.M
106–148	4525.2573	4525.2633	F.MAIPPKKNQDKTEIPTINTIASGEPTSTPTTEAVESTVATLED.S
149–169	2196.1274	2196.1162	D.SPEVIESPPEINTVQVTSTAV.-

^1^ Mr (expt) means experimental molecular weight. ^2^ Mr (calc) means calculated molecular weight. ^3^ MALDI-TOF-MS peptide map of κ-casein hydrolyzed by RmproB.

**Table 3:** Molecular Weight Distribution and ACE-Inhibitory Activity of the Duck Hemoglobin and Hydrolysates ^1^.

Hydrolysis Time (h)	Molecular Weight Distribution (%)				ACE-Inhibitory Rate (%) ^2^
	<1 kDa	1–2 kDa	2–5 kDa	>5 kDa	
0	16.8	2.9	13.5	66.8	8.6 ± 1.2 ^a^
2	71.9	13.5	10.1	4.5	71.9 ± 1.6 ^b^
4	79.8	12.0	7.3	0.9	79.2 ± 0.2 ^c^
6	84.1	10.8	5.1	0.0	85.1 ± 1.2^d^
8	88.5	8.9	2.6	0.0	90.7 ± 1.4 ^e^

^1^ The ACE-inhibitory rate was shown as mean ± SD (n = 3). A value of *p* < 0.05 was considered statistically significant. ^2^ The ACE-inhibitory rate was determined at concentration of 0.5 mg/mL.

## Data Availability

The gene sequence in this study has been deposited to the NCBI GenBank database under the accession number MZ547666.

## Supplementary Materials

The following are available online at https://www.mdpi.com/article/10.3390/foods10122949/s1, Figure S1: Standard curve of tyrosine. Figure S2: Optimal pH (A) and optimal CaCl_2_ concentration (B) for MCA (milk-clotting activity) of RmproB. Figure S3: Molecular weight distribution of the duck hemoglobin hydrolysates at different hydrolysis intervals. Table S1: The sequences of all primers used. Table S2: Purification summary of the recombinant protease (RmproB) expressed in *A. niger*. Table S3: Inhibition of the recombinant protease (RmproB) by various protease inhibitors.
