# Supplementary material for: Characterization of a Novel Aspartic Protease from Rhizomucor miehei Expressed in Aspergillus niger and Its Application in Production of ACE-Inhibitory Peptides
Source: Foods. 2021 Nov 30;10(12):2949. doi: 10.3390/foods10122949 (PMC8701012; doi:10.3390/foods10122949)
Supplement: Supplementary file 1 [file foods-10-02949-s001.zip › foods-1457961-supplementary.pdf]

Characterization of a Novel Aspartic Protease from *Rhizomucor miehei* Expressed in *Aspergillus niger* and Its Application in Production of ACE-Inhibitory Peptides

Shounan Wang [1], Peng Zhang [2], Yibin Xue [1], Qiaojuan Yan [2*], Xue Li [2], Zhengqiang Jiang [1*]

[1] *Department of Nutrition and Health, College of Food Science and Nutritional Engineering, China Agricultural University, Beijing 100083, China*

[2] *Key Laboratory of Food Bioengineering (China National Light Industry), College of Engineering, China Agricultural University, Beijing 100083, China*

[*] *Corresponding authors. Tel.: +86 10 62737689; fax: +86 10 82388508. E-mail: yanqj@cau.edu.cn (Q.J. Yan); zhqjiang@cau.edu.cn (Z.Q. Jiang).*

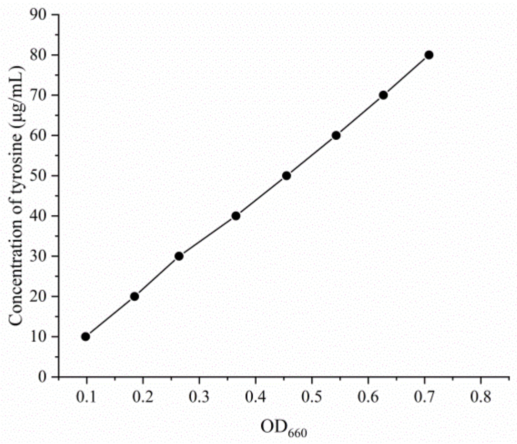

Figure S1. Standard curve of tyrosine. The response was found to be linear in the concentration range of 10~80 μg/mL. The R^2 value was 0.9994, and the regression equation was y=113.34x-0.973. The protease activity (U/mL) = (113.34*OD_{660}-0.973)*0.4.

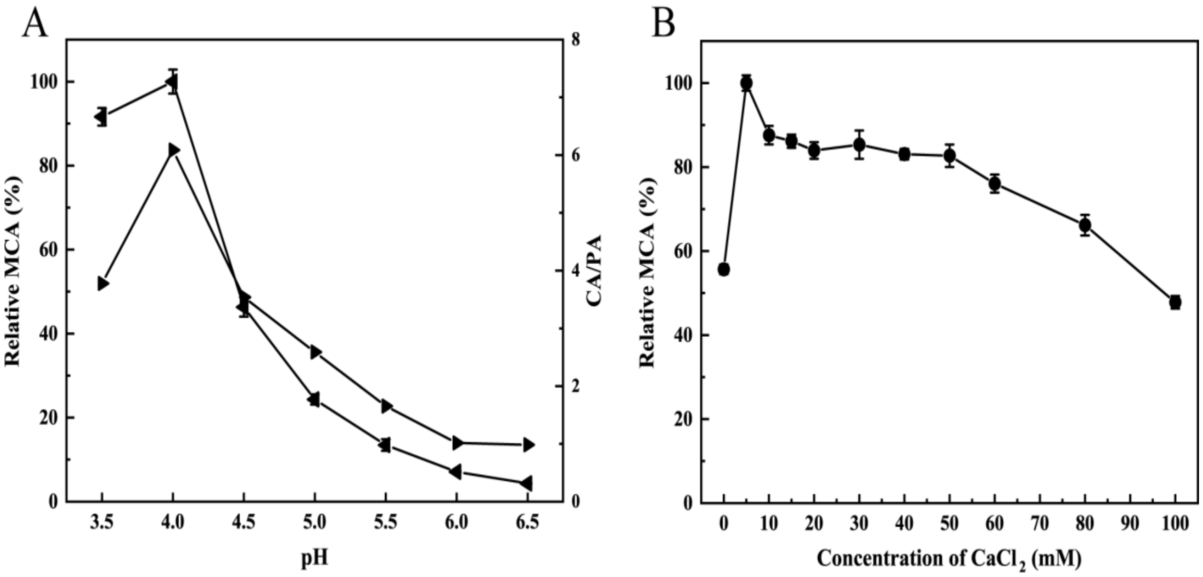

Figure S2. Optimal pH (A) and optimal $CaCl_2$ concentration (B) for MCA (milk-clotting activity) of RmproB. (A) The optimal pH for MCA was determined in 50 mM citrate buffer (pH 3.5–6.5). Symbols: relative MCA (%) (◀); CA/PA (ratio of MCA to protease activity) (▶). (B) The optimal $CaCl_2$ concentration for MCA was evaluated in 10% skim milk powder substrates containing 0–100 mM $CaCl_2$. The milk-clotting activity at 5 mM $CaCl_2$ was taken as 100%.

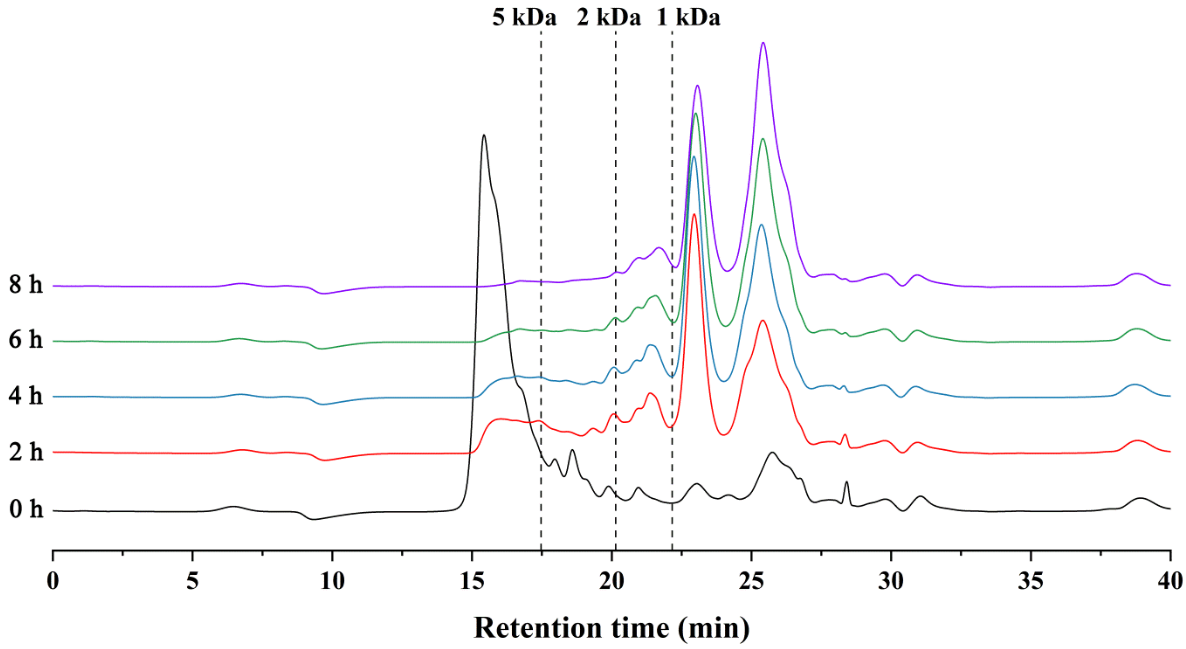

Figure S3. Molecular mass distribution of the duck hemoglobin hydrolysates at different hydrolysis intervals. 0, 2, 4, 6, and 8 h were represented the hydrolysates with corresponding hydrolysis time.

Table S1. The sequences of all primers used

Primers	Sequences
Pgla-RmproB-F	*TCTGCACAGGGTTGGCA*GCTCTCACCAGAATTCCTATCAAGAAGAC
RmproB-TtrpC-R	*ATTTCAGTAACGTTAAGT*CTAGCTTGCGACAATCTGATGTAGATGAA
Pgla-F	CCTCTCGTATGCAGAGGAAATCTCC
Pgla-R	TGCCAACCCTGTGCAGACGAGGCCGCT
Tgla-F	ACAATCAATCCATTTCGCTATAGTTAAAGGATG
Tgla-R	TACCCTTTTCCTTGATTCTCGCCTGCGTA

Table S2. Purification summary of the recombinant protease (RmproB) expressed in *A. niger*

Step	Total activity (U) [1]	Total protein (mg) [2]	Specific activity (U/mg)	Purification (fold)	Recovery (%)
Crude enzyme	32572.9	40.2	810.1	1	100
Q Sepharose Fast Flow	14928.7	6.9	2155.1	2.7	45.8
Sephadex S-100	6139.5	1.9	3176.1	3.9	18.8

[1] Enzyme activity was measured in 50 mM lactate buffer pH 2.5 at 40 °C using casein as substrate.

[2] The concentration of protein was measured by the Lowry method [28].

Table S3. Inhibition of the recombinant protease (RmproB) by various protease inhibitors[1]

Inhibitors	Concentration (mM)	Specific activity (U/mg)	Relative activity (%)
Control	0	3176.1 ± 67.1	100
Pepstatin A	0.01	448.1 ± 29.6	14.1 ± 0.9
	0.05	ND[2]	ND
Iodoacetamide	1	3092.5 ± 98.5	97.4 ± 3.1
	5	3032.2 ± 65.7	95.5 ± 2.1
PMSF	1	3171.5 ± 39.4	99.9 ± 1.2
	5	3099.5 ± 42.7	97.6 ± 1.3
EDTA	1	3101.8 ± 91.9	97.7 ± 2.9
	5	2997.3 ± 42.7	94.4 ± 1.3

[1] Specific activities are shown as mean ± SD (n=3); the enzyme activities of control without any inhibitor are defined as 100%.

[2] ND, no activity detected.
